# The Contribution of Family Planning towards the Prevention of Vertical HIV Transmission in Uganda

**DOI:** 10.1371/journal.pone.0007691

**Published:** 2009-11-02

**Authors:** Wolfgang Hladik, John Stover, Godfrey Esiru, Malayah Harper, Jordan Tappero

**Affiliations:** 1 Global AIDS Program, National Center for HIV, Hepatitis, STD & TB Prevention, Centers for Disease Control and Prevention (CDC), Entebbe, Uganda; 2 Futures Institute, Glastonbury, Connecticut, United States of America; 3 AIDS Control Programme, Ministry of Health, Kampala, Uganda; 4 UNAIDS, Kampala, Uganda; University of Cape Town, South Africa

## Abstract

**Background:**

Uganda has one of the highest total fertility rates (TFR) worldwide. We compared the effects of antiretroviral (ARV) prophylaxis for the prevention of mother-to-child HIV transmission (PMTCT) to that of existing family planning (FP) use and estimated the burden of pediatric HIV disease due to unwanted fertility.

**Methodology/Principal Findings:**

Using the demographic software Spectrum, a baseline mathematical projection to estimate the current pediatric HIV burden in Uganda was compared to three hypothetical projections: 1) without ARV-PMTCT (to estimate the effect of ARV-PMTCT), 2) without contraception (effect of existing FP use), 3) without unwanted fertility (effect of unmet FP needs). Key input parameters included HIV prevalence, ARV-PMTCT uptake, MTCT probabilities, and TFR. We estimate that in 2007, an estimated 25,000 vertical infections and 17,000 pediatric AIDS deaths occurred (baseline projection). Existing ARV-PMTCT likely averted 8.1% of infections and 8.5% of deaths. FP use likely averted 19.7% of infections and 13.1% of deaths. Unwanted fertility accounted for 21.3% of infections and 13.4% of deaths. During 2008–2012, an estimated 131,000 vertical infections and 71,000 pediatric AIDS deaths will occur. The projected scale up of ARV-PMTCT (from 39%–57%) may avert 18.1% of infections and 24.5% of deaths. Projected FP use may avert 21.6% of infections and 18.5% of deaths. Unwanted fertility will account for 24.5% of infections and 19.8% of deaths.

**Conclusions:**

Existing FP use contributes as much or more than ARV-PMTCT in mitigating pediatric HIV in Uganda. Expanding FP services can substantially contribute towards PMTCT.

## Introduction

Mother-to-child HIV transmission (MTCT) is the second largest mode of HIV transmission worldwide, accounting for some 370,000 infections in 2007 [1]. Most of these infections occur in the developing world, particularly in sub-Saharan Africa, where the prevention of mother-to-child transmission (PMTCT) remains a promising, yet complex and challenging programmatic undertaking. In 2007, uptake for antiretrovirals for PMTCT (ARV-PMTCT) reached 33% in sub-Saharan Africa [2]. In many countries PMTCT programs focus on antenatal HIV testing, provision of ARV prophylaxis to HIV-infected women and their newborns, and counseling on safer infant feeding practices. However, these three components constitute just one of four pillars for PMTCT; the remaining being primary HIV prevention in women of child-bearing age, family planning (FP) for the prevention of unwanted pregnancies, as well as care and treatment for HIV-infected women [3] and their HIV-affected children [4].

In Uganda, the HIV epidemic continues to exert a high toll on children. Uganda's goal for 2012 is to reduce MTCT of HIV by 50% [5]. The high burden of pediatric HIV disease, estimated at 120,000–150,000 children living with HIV/AIDS in 2007 [6], stems from a 7.5% HIV prevalence among adult women aged 15–49 years [7] as well as from a relatively low ARV uptake estimated at 39% among HIV-infected ante-natal clinic attendees and 18% among HIV-exposed newborns[8]. Ugandan women, however, also have very high fertility accompanied by a low contraceptive prevalence rate (CPR, estimated at 19.6% [9] among women aged 15–49 years), fueling a population growth estimated at 3.2% annually [10]. Uganda's total population grew from 11 million in 1975 to 24 million in 2000 and is projected to reach 53 million in 2025.[10] Part of this high fertility is due to unintended pregnancies, which, if occurring in HIV-positive women, contribute to thousands of pediatric HIV-infections. Reynolds et al. [11] estimated that more than 7,000 vertical HIV infections in Uganda are averted annually by current contraceptive use. Not surprisingly, strengthening FP as a component of PMTCT [12] has recently received more attention [11], [13]–[15].

In this analysis we used demographic projections to compare the effects of present and projected ARV-prophylaxis and FP use on decreasing the burden of pediatric HIV disease in Uganda, and the contribution of continued unwanted fertility on this burden.

## Methods

### Data sources

The main data sources used for this analysis included the 2006 Demographic and Health Survey [9], the 2004/5 Uganda HIV Sero-Behavioral Survey (UHSBS) [7], and United Nations reports (UN Population Division [10]). We adopted the adult HIV prevalence estimates by the MOH which was generated with the Estimation and Projection Package [16] software. Most input data for this prevalence estimate originated from the ante-natal clinic based HIV surveillance system; the resulting curve was then calibrated by the 2004/5 UHSBS-based HIV prevalence estimate (all adults: 6.4%, females: 7.5%, males: 5.0%). Because the MoH had not released a new national HIV prevalence estimate at the time of this analysis, we assumed the 2005 HIV prevalence to remain constant through 2012. For the ARV uptake for PMTCT (defined as a nationally approved ARV regimen for PMTCT) we used program data to estimate uptake until 2008, when uptake reached 39%. For the years 2009–2012, Uganda has a national PMTCT uptake goal of 80% [5]; however, existing program data and funding environment suggest that this target will not be achievable. Hence the authors adopted a more modest but achievable increase of 4% (absolute) among HIV-infected pregnant women per year in ARV-PMTCT uptake from 2009 to 2012, translating into an ARV-uptake of 57% among ante-natal clinic attendees by 2012. This modest proportional increase nevertheless translates into large increases in absolute numbers, from an estimated 26,600 HIV-infected women receiving ARV-PMTCT in 2007 to 59,500 in 2012. Following national policy goals, we also phased out gradually the use of single dose Nevirapine by assuming that combination ARVs will make up 83% of all ARV regimens by 2010 and 100% by 2012. DHS data suggests a mean breastfeeding duration of 21 months [9]. Other key input parameters such as MTCT probabilities by ARV prophylaxis regimen and infant feeding mode, or total fertility rate [10] (TFR, 6.7 in 2007) are listed in Table 1. The need for cotrimoxazole (CTX) prophylaxis was applied to all HIV-exposed children until 18 months of age, to HIV-infected children under the age of 5 years, as well as to HIV-infected children 5 years or older and not receiving anti-retroviral therapy (ART) [17]. The need for ART was defined as all HIV-infected infants and all children who have progressed to moderate-to-severe HIV disease [17].

**Table 1:** Parameters used in analysis.

***Dynamic parameters***				
	**Year**	**2007**	**2008**	**2012**
Total population (millions) [10]		31.5	32.6	37.7
Total fertility rate (live births per woman life) [9], [10]		6.7	6.7	6.5
without family planning		8.6	8.6	8.3
w/o unwanted fertility		5.1	5.1	4.9
PMTCT uptake (%), total [8] †		33	39	57
Single dose Nevirapine		31	31	0
Dual ARVs		2	7	48
Triple ARVs		0	1	8
CTX (cotrimoxazole) uptake (pediatric)		25%	30%	50%
No. on ART (pediatric) [28]		8,100	12,300	29,400
***Static parameters***				
Fertility ratio [17]		15–19 yrs.	20–49 yrs.	--
HIV-infected:uninfected		1.5	0.47–0.76	--
Breastfeeding [9]		Exclusive	Mixed	Replacement
Distribution by feeding mode		17%	69%	14%
MTCT risk, by feeding and ARV prophylaxis pattern				
*Perinatal* [17], [29]–[31]		*Postnatal (per month)* [17], [32]		
No prophylaxis	20%	ART (taken by mother)		0.3%
Single dose nevirapine	11%	Exclusive BF, 1–6 mths		0.8%
Dual ARV	4%	Mixed BF, 1–6 mths		1.5%
Triple ARV prophylaxis	2%	Mixed BF, 6–36 mths		0.8%
Triple ARV treatment	2%	--		--
Annual survival on ART among [17]	Infants	Children		Adults
1st year	85%	85%		86%
Subsequent years	N/A	93%		90%
Pediatric mortality reduction due to CTX [17]	33%	(First 5 years, w/o ART)		

Footnote: Dynamic data reflect mid-year estimates and may thus differ from cited sources. MTCT: Mother-to-child transmission. ARV: anti-retrovirals. ART: anti-retroviral therapy. BF: Breastfeeding. CTX: cotrimoxazole. N/A: Not applicable.

† For the years 2009–2012, rationale provided in *Methods* */* *Data sources*.

### Data analysis

**Figure 1:**
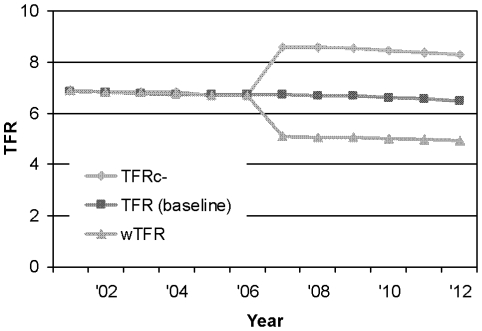
Fertility projections used in analysis. This figure depicts the three different fertility scenarios used in analysis, from 2007 onwards. TFRc-: Total fertility rate in the absence of contraceptive use; TFR (baseline): Current and projected TFR; wTFR: TFR if only wanted births are considered.

Most data analysis was performed with Spectrum [18] (version 3.3), a demographic projection software with a built-in HIV/AIDS module; some Spectrum output data were further analyzed using Microsoft Excel. Using Spectrum, we estimated the actual burden of pediatric HIV/AIDS for the year 2007 and for the period from 2008 to 2012 (baseline projection). The primary outcomes examined included the number of incident pediatric HIV infections, the need for cotrimoxazole and antiretroviral treatment among HIV-infected children aged 0–14 years, and the number of AIDS orphans. We then compared this baseline projection to the following three hypothetical projections: 1) A projection where all ARV uptake for PMTCT was removed, allowing the estimation of the number of HIV-positive births (and subsequent events) averted by ARV prophylaxis. 2) A projection without any FP use, resulting in the higher TFRc- (no contraceptives) and thereby facilitating the estimation of the number of (HIV-positive) pregnancies and subsequent events averted. 3) A projection where all unwanted fertility was removed, resulting in a lower wTFR (wanted TFR), thus allowing the estimation of the actual burden of pediatric HIV disease due to unwanted births. All three hypothetical projections were identical to the baseline projection until the year 2006. From 2007 onwards, the hypothetical projections differed from the baseline projection only in the specific alteration described above. Until the year 2006, all input values were the same for all projections. From 2007 onwards, input values in the different projections varied only to the extent described above. TFR, TFRc-,and wTFR are not specific to the HIV status of women. Based on UN data [10], TFR was projected to slowly decline until 2012 (by approximately 3% between 2007 and 2012, Table 1). TFRc- and wTFR values were both calculated for 2007 using DHS data [9]. To obtain TFRc- and wTFR values for 2008–2012, we applied the 2007 ratio of TFRc-/TFR and wTFR/TFR to the year-specific TFR values, respectively. Hence the three different fertility rates remained constant to each other (Figure 1). The difference between TFRc- and TFR thus indicates the effect of existing (current) and projected (future) FP use, whereas the difference between TFR and wTFR indicates the effect of unwanted fertility ( = unmet need for FP). The difference between TFRc- and wTFR represents the combined total effect of unwanted fertility, partially realized through existing/projected FP use. To estimate the combined effect of contraception and ARV-PMTCT, we compared the baseline projection to a projection where both contraceptive use and ARV-PMTCT services were removed from 2007 onwards.

Using output data from these projections, we also estimated the number of events averted for each 1,000 women exposed to ARV-based PMTCT and/or FP programs. These rates were calculated as follows: Numerators indicated events averted, as shown in Table 2; the denominator for FP was calculated as the proportion of women using FP (CPR 19.6%) times the number of women aged 15–49 [6.7 million], yielding 1,314,000. For ARV-PMTCT, the denominator was calculated as the proportion of HIV-infected pregnant women receiving ARV prophylaxis (estimated as 29.9%) multiplied by the number of total births (1,529,000), yielding 457,000 women. These proportions were then multiplied by 1,000. In a sensitivity analysis, we estimated the effects of an ARV-based PMTCT program that would reach the national PMTCT uptake goal of 80% by 2012 as well as that of a hypothetical FP program targeting HIV-infected women (leaving CPR unchanged for HIV-uninfected women), reaching approximately 50% FP use by 2012. The 50% FP use target represents approximately twice the proportion of HIV-infected persons who are currently aware of their serostatus (based on AIS 2004/5 data indicating that approximately 20% of HIV-infected Ugandan adults had undergone HIV testing in the past). All numerical estimates were rounded to the nearest 100 (for values <10,000) or 1,000 (for values >10,000). All stated ARV-PMTCT uptake figures were adjusted in the analysis for incomplete ante-natal clinic attendance (estimated at 92%, using MOH ANC program and Spectrum projection data for 2007). For the purpose of this analysis, the terms “PMTCT” and “ARV-based PMTCT” are used synonymously unless stated otherwise. We defined children as between 0 and 14 years old, and AIDS orphans as between 0 and 18 years old who have lost at least one parent due to AIDS. Following DHS methods, the term “family planning” includes female/male sterilization, pill, intrauterine devices, injectables, implants, condoms, lactational amenorrhea, emergency contraception, as well as the rhythm and withdrawal method or any “folk method” mentioned by the respondent.

**Table 2:** Estimated effects of ARV-PMTCT, FP, and unwanted fertility on pediatric HIV, 2007.

		Number (%) averted by**)				Number (%) due to unwanted fertility***	
Indicator	Total*	ARV-PMTCT		Family planning			
Births (regardless of HIV status)	1,530,000	0	(0.0)	425,000	(21.7)	371,000	(24.3)
HIV-positive pregnancies	105,000	0	(0.0)	29,000	(21.7)	25,000	(23.9)
Vertical HIV infections (incident)	25,000	2,200	(8.1)	6,100	(19.7)	5,300	(21.3)
No. needing CTX prophylaxis	236,000	100	(0.0)	36,000	(13.3)	31,000	(13.2)
No. needing ART	49,000	2,300	(4.5)	5,900	(10.8)	5,100	(10.5)
Pediatric AIDS deaths (2007)	17,000	1,600	(8.5)	2,600	(13.1)	2,300	(13.4)
AIDS orphans (prevalent)	1,521,000	−1,800	−(0.1)	1,000	(0.1)	600	(0.0)

Note: *The column “Total” indicates the estimated number of persons in the presence of the actual PMTCT and FP uptake.

** The columns “ARV-PMTCT” and “Family planning” indicate the number of events (person characteristic) that would have occurred in their respective absence. Negative values denote increases.

*** The column “Number (%) due to unwanted fertility” indicates how many events (person characteristic) in the “Total” column are due to unwanted fertility.

Percent estimates: for ARV and existing FP, the denominator comprises the total value plus the averted events; for unwanted fertility, the denominator is given by the total value. PMTCT: Prevention of mother-to-child transmission; ARV: antiretrovirals; CTX: cotrimoxazole; ART: Anti-retroviral treatment.

## Results

### 2007 estimates

Table 2 shows the results for the year 2007 when an estimated 1,530,000 births occurred. ARV-PMTCT (2007 uptake: 33%) has no measurable effect on the number of HIV-positive pregnancies occurring in Uganda. Existing FP use likely averted an additional 425,000 births including 29,000 HIV-positive pregnancies whereas unwanted fertility resulted in an estimated 25,000 HIV-positive pregnancies. Approximately 25,000 vertical HIV infections occurred in the presence of the existing FP use and ARV-PMTCT program. PMTCT averted some 8.1% of vertical infections, FP 19.7%, and unwanted fertility accounted for 21.3%. ARV-PMTCT had no substantial effect on the number of HIV-exposed infants needing CTX prophylaxis that same year. FP use reduced the need for CTX prophylaxis by 13.3%; unwanted fertility accounted for a similar proportion. ARV-based PMTCT reduced the number of children requiring ART and the number of AIDS deaths by an estimated 4.5% and 8.5%, respectively. In addition, FP use averted 10.8% and 13.1%, respectively, while unwanted fertility accounted for similar proportions. By keeping more children HIV-uninfected and surviving their HIV-infected mothers, ARV-PMTCT slightly increases the number of AIDS orphans.

### 2008–2012 estimates

Table 3 shows estimates for 2008–2012. During this 5-year period, projected contraceptive use (held stable) is likely to avert approximately 2.4 million births (of any HIV-status); in contrast, unwanted fertility may lead to slightly more than 2 million births. FP use may reduce HIV-positive pregnancies by 22% whereas unwanted fertility is likely to account for almost a quarter of all HIV-positive pregnancies in antenatal care. Compared with 2007, the increase in ARV uptake for PMTCT (from 33% to 57%) and the switch to more efficacious ARV regimens likely will substantially increase the proportion of vertical infections averted (from 8.1% to 18.1%). In comparison, FP use is projected to avert 21.6% of vertical infections whereas unmet FP needs may be responsible for 24.5% of infections, i.e., the met and unmet FP needs combined correspond to 46% of vertical infections. By 2012, the scale up of ARV-PMTCT may avert 24.1% of vertical infections, still falling short of its goal to reduce MTCT by 50%. However, adding existing FP use, which by itself is projected to avert 21.6% of MTCT, together with ARV-PMTCT may avert 40.6% (separate analysis, data not shown) of MTCT, and addressing unmet FP needs would help surpass this goal.

**Table 3:** Estimated effects of ARV-PMTCT, FP, and unwanted fertility on pediatric HIV, 2008–2012.

		Number (%) averted by				Number (%) due to unwanted fertility	
Indicator	Total	ARV-PMTCT		Family planning			
Births (regardless of HIV status)	8,475,000	0	(0.0)	2,353,000	(21.7)	2,053,000	(24.2)
HIV-positive pregnancies	617,000	0	(0.0)	171,000	(21.7)	149,000	(24.2)
Vertical HIV infections (incident)	131,000	29,000	(18.1)	36,000	(21.6)	32,000	(24.5)
Children needing CTX prophylaxis (PY)	1,226,000	12,000	(1.0)	232,000	(15.9)	202,000	(16.5)
Children needing ART (PY)	266,000	37,000	(12.2)	40,000	(13.1)	35,000	(13.2)
Pediatric AIDS deaths	71,000	23,000	(24.5)	16,000	(18.5)	14,000	(19.8)
AIDS orphans (PY)	6,877,000	−55,000	−(0.8)	81,000	(1.2)	67,000	(1.0)

Note: PY: person-years. CTX: cotrimoxazole.

Large differences were observed in the effect of ARV-based PMTCT and FP on care and treatment needs expressed as PY (person-years, Table 3). From 2008–2012, PMTCT will mitigate the estimated need for pediatric CTX prophylaxis and ART by 1% and 12.2% respectively; FP use will reduce these needs by an estimated 15.9% and 13.1%, respectively, and whereas unmet FP needs are responsible for an estimated 16.5% and 13.2%, respectively. ARV-PMTCT may substantially reduce pediatric AIDS deaths (24.5%), more than the met (18.5%) or unmet (19.8%) FP needs will likely reduce or account for. As in 2007, the survival benefit of HIV-exposed newborns provided through ARV-PMTCT continues to paradoxically increase the number of AIDS orphans marginally by 0.3% in 2008 and 1.5% in 2012. FP use probably leads to a small decrease in AIDS orphan years (1.2%, 2008-12).

### Sensitivity analysis

Table 4 shows the hypothetical achievements of an ARV-PMTCT program scaled up to reach 80% uptake by 2012 as well as the potential effects of FP use by 50% of all HIV-infected women aged 15–49 years. Table 5 restricts the results to the last year of the scale-up. In this scenario, the number of vertical infections averted remain below Uganda's goal of preventing 50% of all MTCT-related HIV infections through ARV-PMTCT (Table 5). However, adding the effects of projected FP use (as shown in Table 3) would avert approximately 46% of MTCT, close to the stated goal. Conversely, by focusing FP services on HIV-infected women (50% FP use) together with the more realistic ARV-PMTCT scale up scenario reaching 57% uptake these two interventions combined are estimated to avert 54% of all MTCT in 2012.

**Table 4:** Estimated effects of a 80% ARV-PMTCT uptake or 50% FP use among HIV-infected women, 2008–2012.

		Number (%) avertable by intervention**			
Indicator	Total*	ARV-PMTCT		FP use	
HIV-positive pregnancies	617,000	0	(0.0)	268,000	(34.0)
Vertical HIV infections (incident)	131,000	38,000	(23.8)	55,000	(32.9)
Children needing CTX prophylaxis (PY)	1,226,000	16,000	(1.3)	341,000	(23.4)
Children needing ART (PY)	266,000	49,000	(16.2)	59,000	(19.2)
Pediatric AIDS deaths	71,000	30,000	(32.1)	24,000	(27.0)
AIDS orphans (PY)	6,877,000	−66,000	−(1.0)	53,000	(0.8)

Note: *The column “Total” estimates the number of events or person years as given by the baseline projection, in the presence of the actual estimated uptake of ARV-PMTCT and FP.

** The columns “ARV-PMTCT” and “FP use” indicate the number of events (person characteristic) that would occur in addition in their respective absence. Negative values denote increases.

Percent estimates: the denominator is give by the number in the absence of the intervention; the numerator indicates the number of events averted. CTX: cotrimoxazole. PY: person-years. FP: Family planning. 80% ARV-PMTCT uptake and 50% FP use (among HIV+ women) are scaled up beginning 2009 and reach their goals by 2012.

**Table 5:** Estimated effects of a 80% ARV-PMTCT uptake or 50% FP use among HIV-infected women, by 2012.

		Number (%) avertable by intervention			
Indicator	Total	ARV-PMTCT		FP use	
HIV-positive pregnancies	135,000	0	(0.0)	78,000	(45.2)
Vertical HIV infections (incident)	26,000	12,000	(34.4)	15,000	(44.3)
Children needing CTX prophylaxis	254,000	7,000	(2.7)	102,000	(32.8)
Children needing ART	57,000	16,000	(23.3)	17,000	(25.4)
Pediatric AIDS deaths	12,000	9,000	(47.6)	6,200	(39.7)
AIDS orphans	1,280,000	−25,000	−(2.0)	26,000	(2.0)

Note: All values shown apply to the year 2012 only.

### Events averted per 1000 women exposed to ARV-PMTCT or FP

As outlined in the *Methods* section, we also examined the separate impact of ARV-PMTCT and FP as expressed per 1,000 women exposed to either service; results are shown in Table 6. Both interventions are similarly effective in averting vertical infections although substantial differences can be observed for other indicators.

**Table 6:** Pediatric events averted annually for each 1000 women (any HIV status) utilizing either PMTCT or FP services.

	PMTCT	FP
Births (regardless of HIV status)	0.0	323.4
HIV-positive pregnancies	0.0	22.1
Vertical HIV infections	4.8	4.6
Children needing CTX prophylaxis	0.2	27.4
Children needing ART	5.0	4.5
Pediatric AIDS deaths	3.5	2.0
AIDS orphans	−3.9	0.8

Note: Data pertain to the year 2007. Negative numbers indicate increases. PMTCT: Prevention of mother-to-child transmission; FP: Family planning; CTX: Cotrimoxazole; ART: Antiretroviral therapy.

## Discussion

Every day in Uganda, FP averts approximately 20 vertical infections and 9 pediatric AIDS deaths. Our comparative analysis suggests that existing FP services significantly contribute to the goals of PMTCT in Uganda, exceeding the current achievements of ARV-PMTCT alone. Even the projected expansion of ARV uptake (in scale and quality) from 2008–2012 is matched by the effects of projected use of FP. At the same time, unwanted fertility in Uganda accounts for a substantial amount of pediatric HIV disease and will continue to do so unless access to FP services improves significantly. Targeting FP services for HIV-infected women could have a dramatic positive effect for PMTCT.

Our estimates' validity depends on the underlying formulas used in Spectrum as well as on the validity of the input data and underlying assumptions, such as MTCT probabilities or ARV-PMTCT uptake. The Spectrum software is reviewed by the UNAIDS Reference Group on Estimates, Models and Projections to ensure that it uses the latest data, recreates the pattern of historical epidemics and is consistent with other data sources such as estimates of AIDS mortality [19], the number of orphans and vulnerable children [20], and the number in need of ART [21]. Most assumptions were adopted from the Spectrum software's default values which are based on review of international literature by expert panels. Although ante-natal clinic based HIV prevalence estimates have to be interpreted with caution [22], our estimates mainly rely on a national population-based HIV survey. We kept HIV prevalence stable over time as no new national estimates were available for the years after 2005. Although changes in HIV prevalence would affect the absolute size of estimates (e.g., number of vertical infections), they would have little impact on the relative effects (e.g., proportion of vertical infections averted) of ARV-PMTCT or FP and hence would not change the overall findings and conclusions. TFR was allowed to decline very slowly (as projected by the United Nations) whereas the uptake of combination ARVs steadily rose. The effect of ARV-PMTCT is particularly sensitive to its projected uptake until 2012. As mentioned, we chose a modest but realistic increase in ARV-PMTCT uptake and addressed the international goal of universal PMTCT coverage (corresponding to 80% uptake) in the context of a sensitivity analysis. This is because in order to meet Uganda's programmatic goals (including a reduction of vertical infections by 50% in 2011/2012 compared to 2006/2007), its national strategic plan [5] for 2007–2012 calls for more than a three-fold increase in financial resources. Instead we chose what we thought to be a feasible increase in ARV-PMTCT uptake in an essentially flat funding environment for the foreseeable future. Changes in these and other parameters all affect the outcomes shown here although they would not alter the main conclusions. The inputed MTCT probabilities reflect findings from various trial data, implying that program effectiveness equals trial efficacies. PMTCT reports from Uganda however indicate that perhaps half of HIV-exposed newborns miss taking ARVs for PMTCT even though their mothers receive them [8], a factor we could not account for in our projections. Finally, better national data on FP service provision, access, and use, as well as TFR, childbearing desires, unintended pregnancies, and induced abortions among women at risk for or infected with HIV would have allowed a more refined analysis and a more informed discussion. Hopefully, such data will be become available in the future.

Our analysis on the effects of FP was limited to those relevant for pediatric HIV from 2007–2012. Because we left TFRc- and wTFR projections unchanged for historical years, we did not consider the effect of past contraceptive use or non-use (prior to 2007) on the current or future burden of pediatric HIV/AIDS. In fact, since the start of Uganda's HIV epidemic (assumed to be in 1980), contraceptive use likely averted a cumulative total of 280,000 vertical HIV infections and 180,000 pediatric AIDS deaths, while unwanted fertility led to 220,000 vertical infections and 140,000 pediatric AIDS deaths until 2009 (separate analysis, data not shown). We also did not consider other well described non HIV-specific benefits of FP such as improvement in gender inequality and female empowerment, reduction in overall child and maternal mortality, improving maternal health, as well as (indirectly) mitigating poverty, hunger, and facilitating universal primary education [23]. Neither did we consider mistimed births, i.e., births occurring earlier than desired and easily avertable by family planning and part of the larger group of unintended pregnancies. Improved access to family planning would likely reduce the high maternal mortality (estimated at 550/100,000 live births [24]) and abortion rate in Uganda, estimated at 54 per 1,000 women [25], translating into approximately 300,000 abortions per year, or one in five pregnancies. Elective abortions are illegal in Uganda [25], and thus are often carried out informally with greater risk for the mother.

The contribution of family planning for PMTCT gained substantial momentum in the literature in this decade [11], [13]–[15], [26], [27]. Reynolds et al. [11] recently analyzed this topic for countries receiving funds from the United States PEPFAR (President's Emergency Plan For AIDS Relief) program, including Uganda, with similar conclusions as in our analysis.

The timing of events leading to an HIV-infected child (HIV acquisition by a woman of reproductive age, followed by pregnancy and subsequent MTCT), makes it clear that family planning for PMTCT is entirely complementary to ARV-based PMTCT. Because family planning precedes ARV-based PMTCT in sequential order, reducing unwanted pregnancies alleviates the resource-intensive ante-natal clinic and ARV-based PMTCT services. Uganda's CPR (contraceptive prevalence rate) is estimated at just 19.6% [9] including traditional methods (4.1%); a CPR increase to 34% (or 41% among currently married women) is necessary to reach the total wanted fertility rate of 5.1 that was used in this analysis. Most HIV-infected Ugandan women of reproductive age are unaware of their HIV status and many would opt for FP if they knew their serostatus or had better access to FP. An immediate and targeted measure calls for better – real – integration of FP services into post-natal care for HIV-positive and indeed all sexually active women of reproductive age. However, PMTCT funds have not been available for this purpose in Uganda [8], not least as some donors do not allow for the procurement of family planning commodities. PMTCT training curricula need to address all four PMTCT pillars as should monitoring and evaluation programs reviewing PMTCT services. Voluntary counseling and testing programs actively offering FP services to HIV-infected adults would avert many subsequent unwanted HIV-positive pregnancies. Moreover, given the risk of HIV transmission associated with unprotected sex, the promotion of dual family planning (the combined use of a barrier with a hormonal FP method) would achieve reductions in both vertical and horizontal HIV infections. Lastly, a general investment to expand FP services for all sexually active persons may yield the broadest benefits for HIV-positive and negative women alike, averting transmission in HIV-discordant relationships and unintended pregnancies (irrespective of HIV status) and its many consequences.

Even the planned expansion of ARV-PMTCT uptake to 80% by itself is unlikely to achieve Uganda's goal to reduce MTCT by 50% [5]. Comprehensive PMTCT that includes both ARV prophylaxis and FP services is needed to make such goals achievable. Modern FP methods are safe, cost-effective [11], [13]–[15], and provide substantial benefits towards PMTCT and beyond. These data demonstrate the substantial potential benefit of increasing FP coverage on reducing HIV infections in children in Uganda. Better integration of FP with PMTCT programs would be an important way to achieve this benefit. Donors, policy makers, and program planners need to acknowledge and embrace the real contribution of FP for PMTCT and support its expansion.
